# Measurement of Nanometre-Scale Gate Oxide Thicknesses by Energy-Dispersive X-ray Spectroscopy in a Scanning Electron Microscope Combined with Monte Carlo Simulations

**DOI:** 10.3390/nano11082117

**Published:** 2021-08-20

**Authors:** Thomas Walther

**Affiliations:** Department of Electronic & Electrical Engineering, University of Sheffield, Mappin Street, Sheffield S1 3JD, UK; t.walther@sheffield.ac.uk

**Keywords:** energy-dispersive X-ray spectroscopy (EDXS), scanning electron microscopy (SEM), penetration depth, oxide thickness, gate oxides

## Abstract

A procedure based on energy-dispersive X-ray spectroscopy in a scanning electron microscope (SEM-EDXS) is proposed to measure ultra-thin oxide layer thicknesses to atomic scale precision in top-down instead of cross-sectional geometry. The approach is based on modelling the variation of the electron beam penetration depth and hence the depth of X-ray generation in the sample as a function of the acceleration voltage. This has been tested for the simple case of silica on silicon (SiO_2_/Si) which can serve as a model system to study gate oxides in metal-on-semiconductor field-effect transistors (MOS-FETs). Two possible implementations exist both of which rely on pairs of measurements to be made: in method A, the wafer piece of interest and a reference sample (here: ultra-clean fused quartz glass for calibration of the effective k-factors of X-ray lines from elements O and Si) are analysed at the same acceleration voltage. In method B, two measurements of the apparent O/Si ratio of the same wafer sample need to be made at different acceleration voltages and from their comparison to simulations the SiO_2_ layer thickness of the sample can be inferred. The precision attainable is ultimately shown to be limited by surface contamination during the experiments, as very thin carbonaceous surface layers can alter the results at very low acceleration voltages, while the sensitivity to ultra-thin surface oxides is much reduced at higher acceleration voltages. The optimal operation voltage is estimated to lie in the range of 3–15 kV. Method A has been experimentally verified to work well for test structures of thin oxides on Si-Ge/Si.

## 1. Introduction and Scope

The key device enabling faster and more compact electronic circuits is the metal oxide semiconductor field effect transistor (MOSFET), the electric behaviour of which is controlled by the thin gate oxide that separates the gate contact from the underlying conductive channel. Gate oxides made from SiO_2_ typically have thicknesses down to 1 nm; scaling down further constitutes a technological problem addressed by novel high-*k* dielectrics which are used to replace SiO_2_ [1] or are grown on top of very thin SiO_2_ layers whose thickness can then be reduced further to 0.7 nm [2,3] or even 0.5 nm [4,5]. Measuring SiO_2_ gate oxide thicknesses to atomic scale precision can, in principle, be done using several methods that can be classed into three groups:

(i) dielectric measurements based on capacitance-voltage profiling [6] or ellipsometry [7] which are quick and easy to use. They provide average values of oxide thicknesses integrated over large surface areas and can be used for routine quality control, assuming the dielectric function of the gate oxide is precisely known;
(ii) spectroscopic methods with finite depth resolution, such as X-ray photoelectron spectroscopy [8] or medium energy ion scattering (MEIS) [9,10], where a depth profile can be numerically reconstructed based on modelling the depth penetration as a function of angle and/or energy;
(iii) destructive analysis in cross-section by e.g., depth profiling techniques based on secondary ion mass spectroscopy (SIMS) [11,12,13] or scanning Auger electron spectroscopy [4,14] at nm-scale resolution, or (scanning) transmission electron microscopy (TEM/STEM) that can provide lattice resolution [4,15,16] and chemical analysis at near-atomic resolution [17]. These techniques are slow, expensive and destroy the sample at the point of analysis so they are unsuitable for mass inspection, but they provide the possibility to study specific sites.

An excellent review in the form of a round-robin survey comparing eight different techniques for measuring gate oxide thicknesses on silicon quantitatively highlights the importance of water adsorption and carbonaceous surface layers that explain some systematic offsets between different measurements [18]; however, it does not even consider scanning electron microscopy with energy-dispersive X-ray spectroscopy (SEM-EDXS) which is a standard laboratory technique for quick qualitative compositional analysis of samples.

The energy of the primary electrons defines their interaction volume with the sample, which increases both laterally and vertically with energy. The dependence of lateral resolution on the acceleration voltage has been studied extensively. The penetration depth into the sample also increases with acceleration voltage and so influences the depth in which the majority of X-rays are produced. This has been used in the past to adjust the depth sensitivity of SEM-EDXS and to measure the thicknesses of surface coatings, however, only for relatively thick layers in the range of >0.05 µm, up to several µm [19,20,21]. 

In this study we expand the same principle to the measurement of gate oxide layers which are orders of magnitude thinner than typical hard coatings used for mechanical tool kits or anti-reflective coatings in optics; hence, X-ray count rates will be much lower and both lower acceleration voltages and the effects of external influences such as the build-up of thin contamination layers of the surfaces during data acquisition need to be considered in detail. This is in line with recent developments of dedicated instrumentation and modelling in wavelength-dispersive X-ray microanalysis (SEM-WDX) where it was recently shown that low-energy spectra taken with very bright electron beams under ultra-high vacuum conditions with multiple spectrometers can allow oxide thicknesses down to 10 nm [22] to be measured, while a commercial publication even claims sensitivity down to 2 nm [23].

## 2. Monte Carlo Simulations of X-ray Generation and Detection in a Scanning Electron Microscope (SEM)

For modelling of the electron penetration, multiple electron scattering, X-ray production and X-ray detection in SEM-EDXS [24] as a function of acceleration voltage, the Monte Carlo package CASINO version 2.42 has been used [25]. This code simulates the X-rays generated and detected by a typical solid-state Si-based detector with an atmospheric thin polymer entrance window.

**Figure 1:**
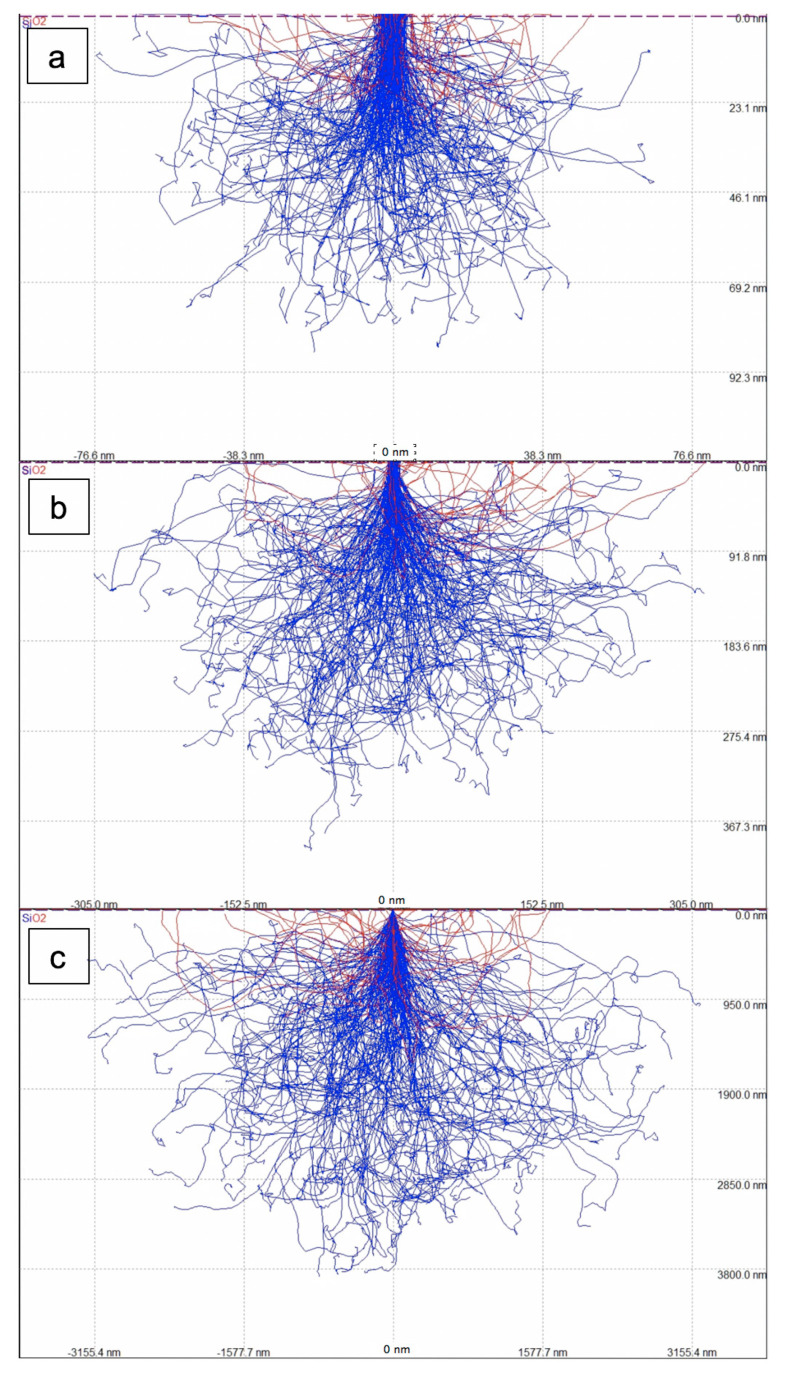
Monte Carlo (MC) simulation by CASINO v2.42 code of electron scattering in 1 nm SiO_2_/Si for three different acceleration voltages of 2 kV (**a**), 5 kV (**b**) and 20 kV (**c**). The trajectories of electrons absorbed in the specimen are shown in blue, those of back-scattered electrons in red. The electron beam impinging vertically was 10 nm in diameter in all cases. Note the different scales: lateral widths are 192 nm (2 kV), 765 nm (5 kV) and 7900 nm (20 kV).

**Figure 2:**
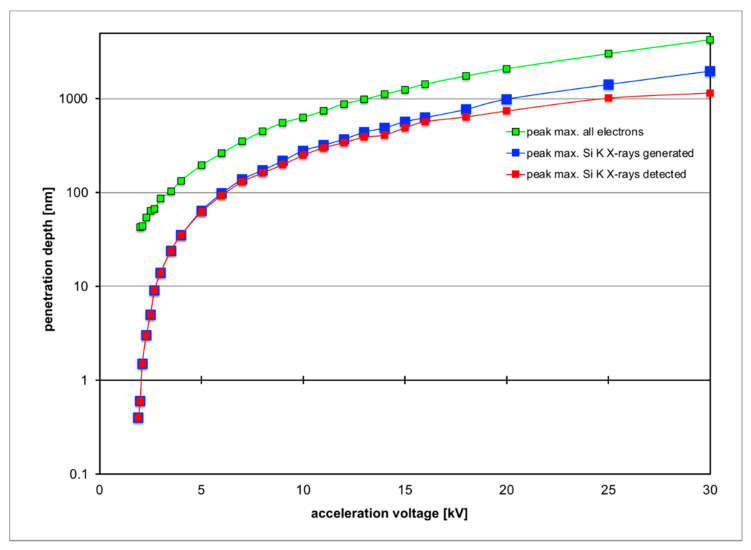
Plots of peaks of maximum penetration depth of primary electrons (green) and Si K-line X-rays generated (red) or detected above the sample (blue).

**Figure 3:**
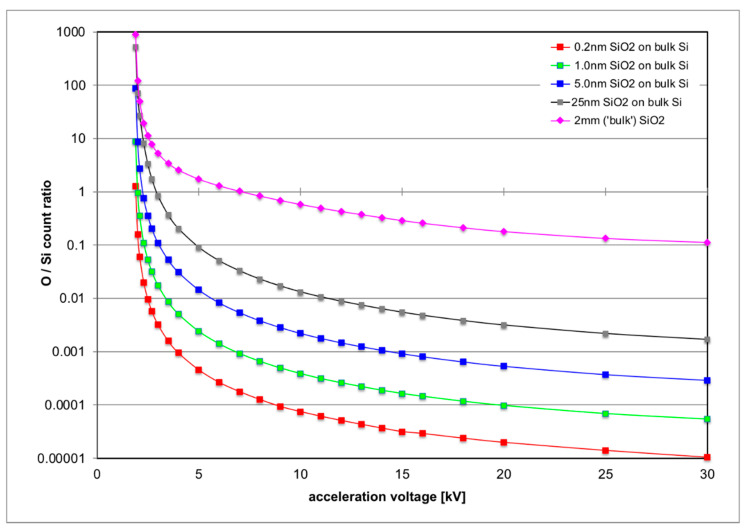
MC simulation of predicted O/Si X-ray count ratio vs acceleration voltage for energy-dispersive X-ray spectroscopy in a scanning electron microscope (SEM-EDXS) with standard Si detector (atmospheric thin film (SATW) polymer window, take-off angle (TOA) of 22°).

For the investigation of SiO_2_ thin layers on Si, the X-ray lines of O K (0.525 keV) and Si K (K_α_ at 1.740 and K_β_ at 1.836 keV) need to be excited, as both intensities are needed: the first for obtaining a signal from the oxide layer itself and the second to serve as a reference signal to compare the other signal to. This means the minimum voltage that can be used as acceleration voltage is 1.9 kV, which just exceeds the ionisation threshold energy for the Si K-edge. The X-ray energy of the Si K_α_ line is simply the difference between ionisation energies for the K-edge at 1.839 keV and the L-edge at 99 eV. At low energies around 2 keV most electrons penetrate about 40 nm into Si and most of the X-rays are generated at a depth of around 1 nm, making such a set-up highly surface sensitive (see Figure 1a). At higher voltages, the electrons become faster, can penetrate further into the material and produce X-rays at greater depth, as shown in Figure 1 and Figure 2. For electron energies above 5 keV, many X-rays are produced at a depth of >60 nm so that their self-absorption in the sample becomes relevant, and the peak depth of X-rays generated (blue curve in Figure 2) is even greater than the peak depth of X-rays detected for a given detector take-off angle (red curve in Figure 2). From such simulations one can, therefore, calculate the expected intensity ratios of measured O/Si X-rays for different geometries, where Figure 3 depicts plots (on a logarithmic scale) for four oxide layers of different thicknesses of 0.2, 1, 5 and 25 nm over the whole range of acceleration voltages from 1.9 to 30 kV. These plots show three distinct properties:

(i) The predicted O/Si ratio scales almost linearly with SiO_2_ layer thickness so that a measurement at a single acceleration voltage would be sufficient to deduce the SiO_2_ thickness if all detector properties (entrance window, top contact and total thickness, all of which determine the detection efficiency) as well as the take-off angle were correctly modelled and if there were absolutely no surface contamination.
(ii) At very low acceleration voltage, the O/Si ratio could be high because both the penetration of the electron beam would be low, picking up a lot of the signal from the oxide layer, but the excitation of the Si K signal would be weak because the electron energy would be only very slightly above the corresponding ionisation threshold. Again, surface contamination will change this somewhat (see discussion later at the end of Section 2 and Section 3).
(iii) For increasing acceleration voltage, the electron beam penetrates further into the sample and picks up more signal from the Si substrate compared to the thin SiO_2_ surface layer, the predicted O/Si ratio decreasing exponentially with energy. As a result, the sensitivity of the signal to the surface would be too low in an experiment at maximum operation voltage to give sufficient count rates from the O K-line to allow for meaningful quantification.

**Figure 4:**
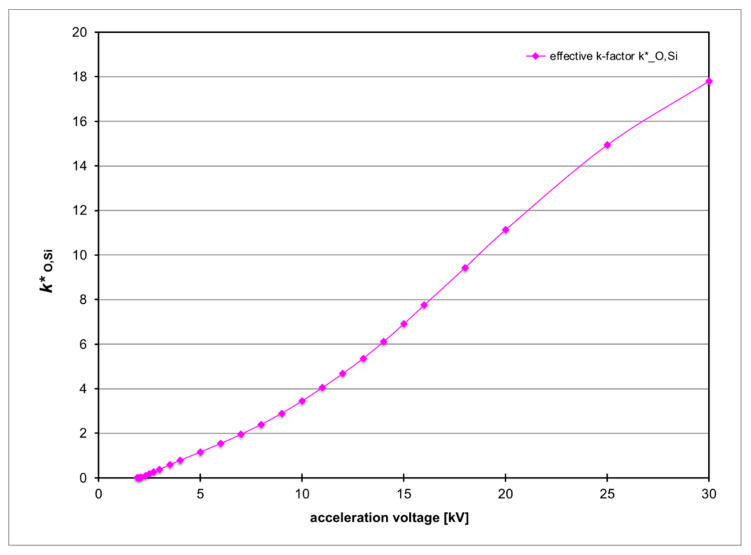
MC simulation of predicted k*_O,Si_ k-factor vs acceleration voltage for SEM-EDXS with standard Si detector (atmospheric thin film polymer window, take-off angle of 22°).

Also shown in Figure 3 is a corresponding plot for a pure SiO_2_ bulk sample (purple diamonds) that shall serve as reference for calibrating the O/Si line intensities. Figure 4 plots the product of corresponding thin-film k-factor for the relevant lines and their absorption correction factor, which can be called an effective *k*-factor k*_O,Si_ [26]. For pure SiO_2_ this is given by the ratio:

(1) $$k_{O,\mathrm{Si}}^{*}\;=\;2I_{\mathrm{Si}}/I_{O}$$

where *I* denotes the intensity of the corresponding X-ray line [27]. This ratio is voltage dependent but no longer thickness dependent as the simulation of bulk here implies infinite thickness of *t* = ∞.

**Figure 5:**
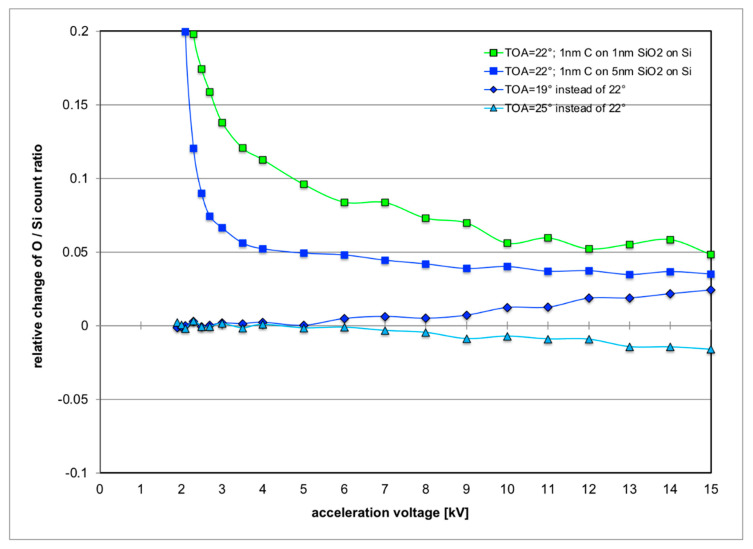
MC simulation of predicted relative change of O/Si X-ray count ratio vs acceleration voltage for SEM-EDXS with standard Si detector when either the take-off angle (TOA) is varied by ±3° or a 1 nm thin carbon contamination of the sample surface is taken into account.

**Figure 6:**
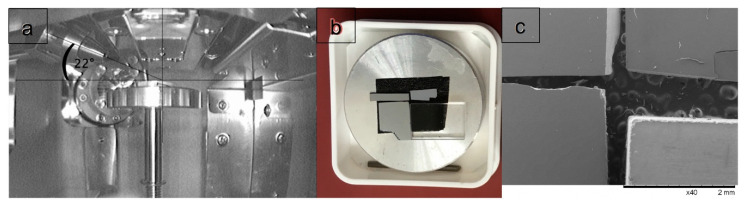
Side view showing SEM chamber with X-ray detector and take-off angle measurement (**a**), image of four specimens mounted on aluminium stub (**b**) and secondary electron image at 15 kV, 40× magnification (**c**) in the Hitachi SEM 3030plus.

Figure 5 investigates how the O/Si ratio expected in a measurement will vary (here plotted as relative change to make plots for 1 nm and for 5 nm SiO_2_ oxide layers comparable) with either the take-off angle (TOA) varied or for contamination in form of a thin surface layer of 1 nm carbon. The motivation for the TOA investigation is that the nominal TOA from the manufacturer specifies 25° while we have actually measured a value of 22° as can be seen from Figure 6a. While its influence at low acceleration voltages is negligible because the low penetration depth means all X-rays come from near-surface regions so self-absorption of X-rays in the sample is irrelevant, its effect increases with acceleration voltage and penetration depth. The overall effect, however, is rather small: ±3° in TOA will change O/Si signals by ±3% at 15 kV at the most. The influence of the surface contamination layer is stronger. The relative changes of the O/Si ratio can be 20% at 2.2 kV and decrease to 3–5% at 15 kV in the simulations. It is perhaps interesting to note that the O/Si ratio is always predicted to go up. 

## 3. Energy-Dispersive X-ray Spectroscopy Measurements in SEM Using Method A

In order to enable sets of coherent experiments under exactly the same conditions, four specimens were mounted onto a common stub for investigation in a Hitachi (Hitachi High Technologies, Tokyo, Japan) tabletop SEM 3030 plus at *U* = 15 kV, a TOA of 22° and magnification of 40×. The microscope is equipped for EDXS with a 30 mm^2^ Bruker (Bruker Nano Analytics, Berlin, Germany) XFlash 430 silicon drift detector that is nominally 450 µm thick, has a 0.029 mm dead layer, an AP3.3 polymer entrance window and provides 126 eV full width at half maximum resolution at 5.9 keV and a sampling of 10 eV/channel.

**Figure 7:**
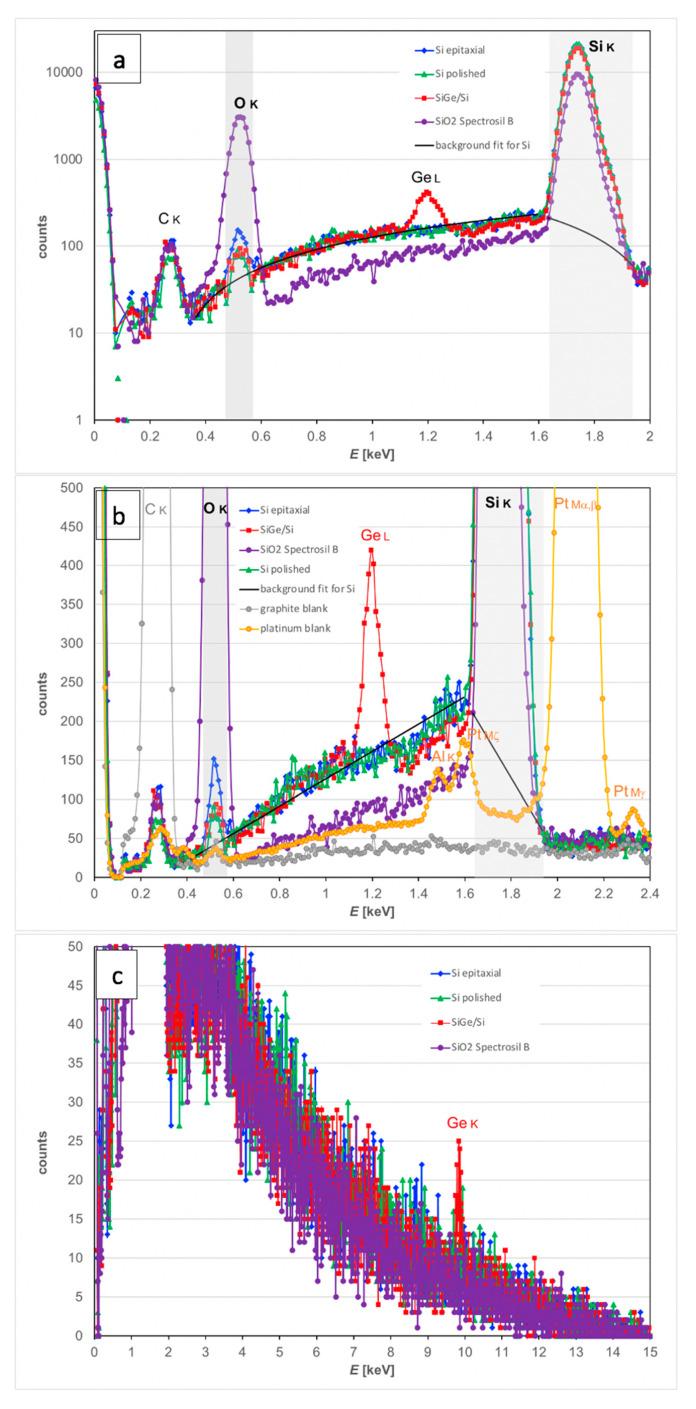
EDX spectra of four specimens at 15 kV: epitaxial Si with 10-year-old native oxide, mechanically polished Si(001) wafer, surface of chemical vapour deposited SiGe 22C86 specimen (cf Figure 8) and fused quartz glass specimen Spectrosil B ©. (**a**) spectral range 0–2 keV on logarithmic scale; (**b**) same spectra as before on linear scale over 0–2.4 keV, with additional data for C and Pt blanks; (**c**) same spectra range on magnified linear scale over 0–15 keV, demonstrating Bremsstrahlung extending out to 15 keV limit even for the silica sample (purple), which showed no charging.

**Figure 8:**
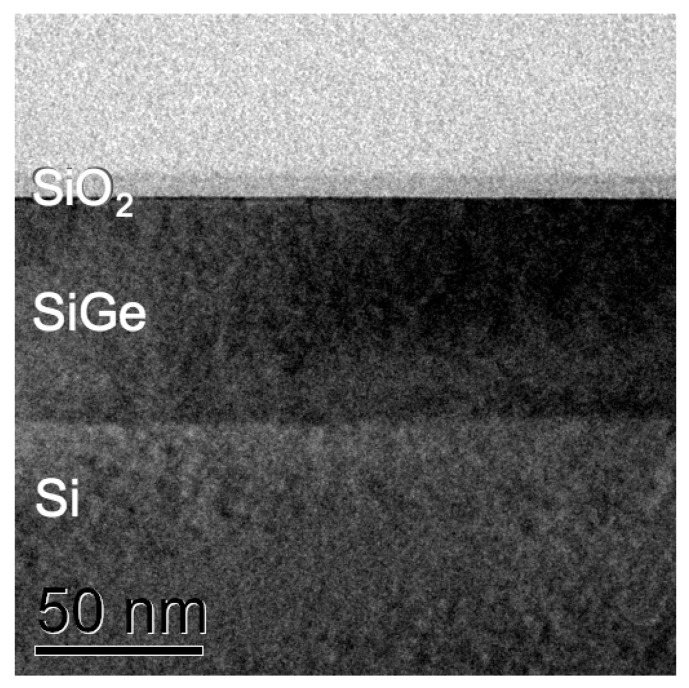
Bright field transmission electron microscopy (TEM) image of sample 22C86, showing from top in cross-section: glue line/6.5 ± 0.3 nm SiO_2_/66.5 nm Si_0.83_Ge_0.17_/Si(001).

The surfaces of all specimens were subsequently cleaned in 99.8% pure ethanol and acetone. No conductive coatings were applied to any sample as none of them showed any signs of charging. This can be seen in Figure 7c where the Bremsstrahlung background of all spectra clearly extends out towards the Duane–Hunt limit at 15 keV in much the same way. The specimens in Figure 6b,c are, clockwise and starting at the top right: a SiGe specimen of known structure that had been previously studied by cross-sectional TEM [28] and the (Si,Ge)O_2_ layer thickness on top of which is known to be now 6.5 nm thick from Figure 8, a 2 mm thick piece of fused quartz glass of type Spectrosil B that serves as an ultra-pure calibration specimen of composition SiO_2_ (trace elements < 10 ppm), a Si wafer piece that had been polished an hour before inspection using 4000 grit silicon carbide paper so the newly formed native oxide on top should be only 1 h old and rather thin, and a piece of Si wafer left over from a previous project that ended in 2011, providing a native oxide on top that was 10 years old.

X-ray spectra were collected at around 10,000 counts per second for 300 s (for Spectrosil B ©: 400 s) while keeping the electron beam scanning regions ≈ 1 mm in diameter in mapping mode to avoid strong contamination often observed otherwise for stationary electron probes or at high magnifications. The resulting spectra are shown in Figure 7. In Figure 7a we concentrate on the energy range up to 2 keV (above that only the SiGe specimen gave a weak Ge K X-ray signal which is irrelevant for our method here). The vertical axis shows the counts on a logarithmic scale so that the rather faint signals of C K, O K and Ge L can be seen before the much larger Si K-line at 1.74 keV. For integration, windows of 475–575 eV are used for the O K-line centred at 525 eV and 1635–1935 eV to include the Si K_α_-line at 1740 eV and K_β_ at 1836 eV. These windows are marked as bars in light grey in Figure 7a,b. The background is fit linearly over 0.3–1.6 keV around but excluding the O K-line itself and 1.6–2.0 keV around but excluding the Si K-line. The fits are shown for the SiGe and Si specimens as black curves in Figure 7; their apparent curvature in Figure 7a is only due to the logarithmic scale of the plot. Then net counts have been extracted by background interpolation and subtraction. These are reported in Table 1, along with additional X-ray measurements conducted for 99.9% pure elemental standards of graphite and platinum that serve as blank controls as they should be free of any surface oxides when prepared in the cleanest possible way (cleaning as before, plus a subsequent anneal at 250 °C for two hours to desorb any solvent residues).

The O/Si ratio of 0.244 measured for the Spectrosil B © sample is in reasonable agreement with the simulated value of 0.289 from the 15 kV value of the top curve in Figure 3, indicating that measured and simulated intensities will not be too far off. Indeed, using the quantification of the *k*-factors built into Bruker’s Quantax 70 software yields a measured atomic ratio of O/Si = 1.834 instead of 2, which is (only) 8% too low. The measured O/Si ratio for the surface oxides of the Si(Ge) wafers is highest for the 10-year-old epitaxial Si wafer, lowest for the freshly polished Si wafer, and lies somewhere in between for the SiGe wafer. 

The physical origin of the small O K-line background (*bg*) signal visible in all spectra, including the graphite and platinum blanks, is presently still unclear: it could be an artefact from the polymer entrance window of our X-ray detector (which would be supported by the correlated increase of spurious C and O counts in all Si wafer samples and the Pt blank) or it could be due to residual hydrocarbon contamination of the surfaces (which would be supported by the observation that even the graphite sample shows some O K-line signal, which would be hard to explain via fluorescence as the C K-line has too low energy for this). It is possible that both effects contribute. Using the four values from the calibration curves for clean Si at 15 kV in Figure 3, fitting a third order polynomial to them and then inverting the relationship numerically, the SiO_2_ thicknesses corresponding to the above background subtracted O/Si ratios in the last column of Table 1 would be estimated as 12.9 nm for the epitaxial Si, 1.7 nm for the recently polished Si wafer and 5.3–6.1 nm for the SiGe wafer where the lower thickness value is obtained if we assume that the oxide on top is pure SiO_2_-on-Si and the upper, more realistic, value if we assume a mixed (Si,Ge)O_2_ oxide on top of SiGe/Si as can be inferred from its influence on annular dark field scanning TEM (ADF-STEM) imaging of similar SiGe layers [29].

However, there are two further points to consider, namely that of statistical and systematic errors. 

**Figure 9:**
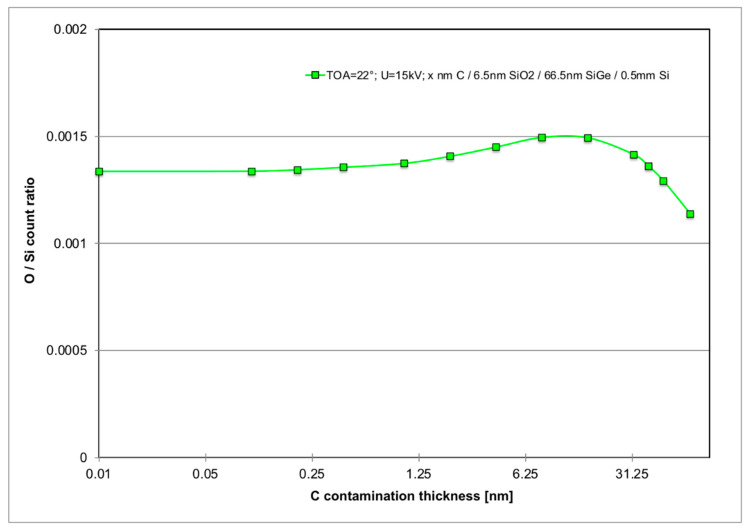
MC simulation of predicted relative change of O/Si X-ray count ratio vs carbon contamination layer thickness *x* in SEM-EDXS (*U* = 15 kV, TOA = 22°), for structure of sample 22C86 as shown in Figure 8 of *x* nm C/6.5 nm SiO_2_/66.5 nm Si_0.83_Ge_0.17_/Si(001). Peak is reached for *x* = 8–16 nm. Mean ± rms value for C thicknesses up to 50 nm: (1388 ± 65) × 10^–6^.

Statistical errors are mainly due to the low count rates for the O K-line intensities, as shown in Table 1. Generally, for a count rate of *N* X-rays, Poisson statistics will give a scatter of √*N*, and errors in background estimation will be of the same order but independent from the above so that the rms error expected will be given by √(2*N*), giving relative errors of √(2/*N*). For the polished Si sample with the thinnest native oxide, a net count rate of *n* = 134 – 81 = 53 will give a relative error of 19% for the oxide thickness, in this case of 1.7 nm, i.e., ±0.3 nm. For the SiGe wafer, *n* = 244 − 81 = 163 gives 11% error in ≈6 nm, i.e., ±0.7 nm; and for the old epitaxial Si sample, *n* = 581 − 81 = 500 gives 6% error in the estimated SiO_2_ thickness of ≈13 nm, i.e., ±0.8 nm. This demonstrates standard deviations of sub-nm in all three cases. A (hypothetical) 1 nm SiO_2_ layer would only yield about 32 counts above the blank level under these conditions and thus have an rms error bar of ±0.25 nm attached to it. A straightforward way to improve statistics would be to extend the measurement time, e.g., to 30 min to get down to ±0.1 nm rms, however, that would also lead to an increase in contamination related to the following point, which in turn will change the measured O/Si ratio slightly, as shown in Figure 9.

Systematic errors are expected due to further carbon build-up on the surface during the measurements even for initially perfectly clean material [30]. Figure 5 shows that already 1 nm of C will increase the measured O/Si ratio slightly (by 3–5% at 15 kV); and Figure 9 below shows (for the specific sample geometry of our SiGe specimen) that this increase is maximal if the C contamination layer thickness is slightly thicker than the SiO_2_ layer thickness to be measured. Comparison of the spectra in Figure 7 and inspection of Table 1 shows that in all Si and SiGe wafers examined here, this has probably been the case. In fact, for the SiGe specimen, the inferred oxide thickness of 6.1 ± 0.7 nm from SEM-EDXS is in agreement with the direct measurement by cross-sectional TEM of 6.5 ± 0.3 nm reported in Figure 8.

## 4. Conclusions

Two methods to measure the thickness of thin SiO_2_ oxide films on Si wafers in plan-view (top-down) geometry by SEM-EDXS have been developed and simulated numerically by Monte Carlo methods. 

Method A based on two measurements at a medium acceleration voltage of the O/Si ratio of both the wafer of interest and a pure SiO_2_ bulk reference specimen has been tested experimentally at 15 kV for three different wafers and delivered results for oxides 1.7, 6.1 and 13 nm thick where TEM independently confirmed the thickness for the 6–7 nm thin layer. For an ultra-thin oxide in the 1 nm range the specimen would have to be transferred from the growth to the SEM vacuum chamber very quickly, possibly using a glove box container with inert gas atmosphere, in order to prevent any further oxidation during transfer, and the acquisition time for EDXS would have to be increased from the 5–6 min used here to almost 1 h (using an SEM with similar beam current and X-ray detection efficiency) in order to obtain sufficient count rates for the O K-line. This is where the advantage of SEM-WDXS with field-emission gun, ultra-high vacuum chamber and multiple X-ray detectors as reported in [22,23] lies compared to SEM-EDXS. 

It is also planned to verify the above measurements using method B; this necessitates an SEM-EDXS instrument that can be operated at various acceleration voltages between ideally 3 and 15 kV, while the Hitachi Tabletop SEM 3030plus model used here only operates at 5 kV and 15 kV but does not allow the use of the X-ray detector at 5 kV. Also, ideally, a windowless X-ray detector should be used to avoid the uncertainty in the origin of the O K-line background observed in all samples here.

## Figures and Tables

**Table 1:** Numerical results for intensities measured from spectra in Figure 7. Colour code correlates with those of spectra in Figure 7. The O K counts from the graphite and platinum standards used as blanks in Figure 7b amount to 75 and 87 counts, respectively, yielding on average an apparent oxygen background signal, *bg* = 81 ± 6 counts, for the O K-line at 300 s.

Specimen	C K Counts	O K Counts	Si K Counts	O/Si Ratio	(O–*bg*)/Si Ratio
**Si epitaxial**	639	581	184,770	0.003144	0.002706
**Si polished**	398	134	188,290	0.000712	0.000281
**SiGe/Si**	586	244	165,749	0.001472	0.000983
**SiO_2_ Spectrosil B**	537	20,036	82,121	0.243981	0.242995
**graphite**	42,290	75	-	-	-
**platinum**	382	87	-	-	-
